# Comparative Analysis of Clinical Trials of Biologic Drugs for Patients with Primary Sjögren’s Syndrome

**DOI:** 10.3390/jcm15030950

**Published:** 2026-01-24

**Authors:** Carlota Navarro-Joven, Silvia Piunno, Maryia Nikitsina, Carmen San José Méndez, David A. Isenberg

**Affiliations:** 1 Department of Rheumatology, Hospital Universitario Puerta de Hierro Majadahonda, 28222 Majadahonda, Spain; carlotanavarroj@gmail.com; 2 Department of Rheumatology, Fondazione Policlinico Universitario Agostino Gemelli IRCCS—Università Cattolica del Sacro Cuore, 00168 Rome, Italy; silviapiunno95@gmail.com; 3 Department of Rheumatology, Hospital Universitario La Princesa, 28006 Madrid, Spain; maryianikitsina95@gmail.com; 4 Department of Rheumatology, Complejo Hospitalario Universitario de A Coruña (CHUAC), 15006 Galicia, Spain; carmensjm23@gmail.com; 5 Department of Ageing, Rheumatology and Regenerative Medicine, University College London, London WC1E 6JF, UK

**Keywords:** primary Sjögren’s syndrome, biologic therapy, clinical trials, systematic review

## Abstract

**Background/Objectives:** To evaluate and compare the characteristics of clinical trials (CTs) involving patients with primary Sjögren’s syndrome (pSS), using biologics, and focusing on the features of the patients recruited. **Methods**: This systematic review assessed pSS CTs evaluating biologic drugs published from 2010 to 2024 according to the Preferred Reporting Items for Systematic reviews and Meta-Analyses (PRISMA) guidelines. The literature search of the electronic databases was performed individually by the authors. The extracted variables regarding the baseline characteristics of participants and trial-related information were defined a priori, collected, and compared. **Results**: A total of 16 CTs were included in this review in line with the inclusion criteria. The trials were predominantly multicenter (75%) randomized controlled trials with a placebo arm (93.8%), with only five trials recruiting participants across multiple (≥3) continents. The search included a total of 1607 patients (mean age 51 years, 94% female) with a mean disease duration of 6.47 years. Race and ethnicity were underrepresented variables, found in 37.5% and 12.5% of the trials, respectively, with White patients comprising the majority (77.8%). The EULAR Sjögren’s Syndrome Disease Activity Index (ESSDAI) was reported in 93.8% of the CTs. However, only recent studies have emphasized it as the primary outcome. **Conclusions**: Recent trials on biologics in pSS patients show better methodological quality, with a more standardized assessment of disease activity using ESSDAI, and an increased focus on patient-reported outcomes. Global participation is increasing, but limited racial and ethnic diversity, endpoint variability, and inconsistent biomarker reporting remain critical issues.

## 1. Introduction

Sjogren’s syndrome (SS) is an autoimmune rheumatic disease characterized by exocrine glandular lymphocytic infiltration causing severe dryness primarily of the mouth and eyes [1]. It predominantly affects the exocrine glands, but in about 30–40% of patients it can have systemic manifestations involving the joints, skin, lungs, kidneys, and peripheral and central nervous system [2]. It can be classified as primary or secondary, based on whether it occurs independently or with other systemic autoimmune diseases, respectively [3]. Currently, there are no disease-modifying pharmacological treatments specifically approved for SS. As a consequence, therapeutic strategies often rely on data extrapolated from the treatment of other autoimmune rheumatic diseases, notably systemic lupus erythematosus (SLE), given their clinical and pathophysiological similarities [4]. The lack of approved therapies has prompted clinical trials (CTs), especially randomized clinical trials (RCTs), aimed at identifying effective treatment options. It must be recognized that there are several challenges when developing potential treatments for SS; notably, its heterogeneous nature, a lack of correlation between symptoms and disease activity, and the significant burden of subjective symptoms such as fatigue and pain with a high response to placebo [5,6].

Given the lack of approved disease-modifying treatments for SS and the limited capacity of standard therapies to alter the underlying autoimmune process, biologic agents are a promising strategy, selectively targeting central immunopathogenic pathways. Advances highlighting the importance of B-cell activation, T-cell help, and cytokine imbalance have led to the development of agents designed to interfere with these mechanisms. Most therapies evaluated in clinical trials act by suppressing B-cell hyperactivity or by modulating T-cell co-stimulation and cytokine networks. Collectively, these targeted approaches aim to modulate the immune pathways dysregulated in SS [1].

We have undertaken a systematic review of CTs involving patients with primary SS (pSS), utilizing biologics, and published between 2010 and 2024, with the aim of identifying potential limitations in study design or data gaps that may contribute to the currently insufficient development of effective therapies for pSS.

## 2. Materials and Methods

### 2.1. Data Sources and Search Strategy

This systematic literature review is conducted in accordance with the Preferred Reporting Items for Systematic Reviews and MetaAnalyses (PRISMA) guidelines for systematic reviews [7] (see Supplementary Materials Files S1–S2 for the PRISMA checklist). The review of the literature was performed in January 2025 using the following electronic databases: PubMed, EMBASE, EBSCO and ClinicalTrials.gov. Keywords in the advanced search included “Sjögren” AND “trial” in the title and abstract. No language restrictions were selected. The research included all phase II and III CTs evaluating biologic treatments and involving at least 30 adult patients (>18 years old) affected by pSS and published from 2010 and 2024. Within this period, CTs were further categorized into those published before 2016 and after 2017. Trials including participants with secondary Sjögren’s syndrome were excluded. Phase 1 CTs, trials evaluating non-pharmacologic interventions, local therapies, conventional immunosuppressive or immunomodulatory drugs, and studies derived from secondary analysis were excluded. Because the terms “biologic” or “biological” are not consistently used in the titles or abstracts of trials evaluating these agents, the search strategy was intentionally kept broad to maximize sensitivity. Eligible biologic trials were subsequently identified during the manual title and abstract screening. Two of the authors reviewed the titles and abstracts resulting from this search, verifying that the publications adhered to the aforementioned eligibility criteria. Discrepancies were resolved by discussion and, in the case of persistent discrepancies, a third author was asked to adjudicate. Duplicates were removed manually.

### 2.2. Data Extraction

Data were extracted by the authors independently, considering the publications and the Supplementary Materials. Each CTs arm was considered separately. Extracted data included demographic characteristics (age, sex, race, and ethnicity); disease-related details including laboratory tests anti-nuclear antibodies (ANA), antibodies against Sjögren’s Syndrome A (anti-SSA) or anti-Ro, antibodies against Sjögren’s Syndrome B (anti-SSB) or anti-La, Rheumatoid Factor (RF), Immunoglobulin G (IgG), Complement (C3 and C4); β2-microglobulin levels, and presence of cryoglobulinemia; the salivary gland focus score (SGFS) including stimulated and unstimulated salivary flow (SSF/USSF); the affected domains; clinical indices covering the EULAR Sjögren’s Syndrome Disease Activity Index (ESSDAI), the EULAR Sjögren’s Syndrome Patient Reported Index (ESSPRI), numerical rating scale (NRS) for dryness, pain, and fatigue, the Mental and Physical Component Summary (MCS, PCS) of Short Form 36 (SF-36), and the Multidimensional Fatigue Inventory-20 (MFI-20); concomitant treatments (the duration of the disease and symptoms); trial-related information (authors, year of publication, sponsor, phase, arms, participants enrolled and completing the trial, inclusion criteria, drug evaluated with mechanism of action, treatment regimen, duration of follow-up, primary and secondary outcomes); and the geographical representation (the number of centers and continents involved and the Human Development Index (HDI) of the included countries). HDI is an index utilized to measure and compare the level of human development among different countries, assessing three fundamental dimensions: health, education and standard of living. Each country with enrolling sites was categorized based on its HDI (very high > 0.8, high 0.7–0.79, medium 0.55–0.69, or low < 0.55) [8]. Discrepancies in data extraction were resolved through discussion to reach consensus, and, if necessary, were adjudicated by a third author.

### 2.3. Risk of Bias Assessment

No risk of bias analysis was required, as no data on trial results were obtained. This review is designed on baseline patient assessment of the CTs included in the analysis. However, potential sources of design and recruitment-related bias were qualitatively assessed, focusing on variability in inclusion criteria, sample size, geographical representation and the completeness of baseline data reported, as these factors could influence the interpretation of the populations enrolled in these trials.

### 2.4. Data Synthesis and Statistical Analysis

Continuous variables were presented as mean (standard deviation [SD]) or median (min or max). Categorical variables were reported as absolute numbers and percentages or proportions.

## 3. Results

**Figure 1:**
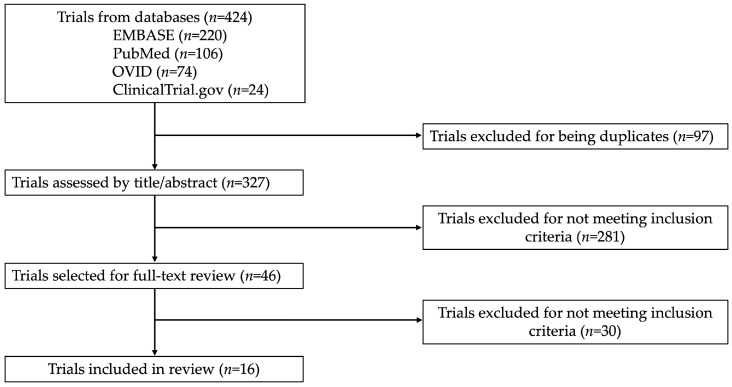
Search strategy flowchart.

The search resulted in 424 records from title and abstract screening. Based on the inclusion criteria and after excluding duplicates, 46 were selected for a full-text review by the authors. Among them, 30 CTs were excluded because, upon reviewing the full-text, the inclusion criteria were not fulfilled, leading to 16 articles being included (Figure 1).

A summary of the main characteristics of the CTs selected is reported in Table 1.

### 3.1. Trial Characteristics and Design

#### 3.1.1. Publication Trends and Sponsorship

This systematic review has analyzed 16 CTs, covering publications from 2010 to 2024 [9,10,11,12,13,14,15,16,17,18,19,20,21,22,23,24]. Most of the trials were conducted in the latter half of this period, with 12 (75%) trials published after 2017.

The funding sources varied considerably although there is a predominance of industry-sponsored trials (*n* = 9, 56.3%), while four (25%) CTs were funded by academic institutions. Hybrid funding models involving academic institutions and industry accounted for two (12.5%) CTs, and one trial was supported by a combination of a government and an academic institution.

#### 3.1.2. Study Phases and Design

Almost all CTs (*n* = 15, 93.8%) were RCTs with a placebo arm, while only one was an open-label trial [11]. The number of arms ranged from two to four: seven CTs (43.8%) had two arms with a 1:1 randomization scheme, while one trial [24], had a basket design, including a four-arm design (1:1:1:1) in the dose-ranging cohort and a two-arm design (1:1) in the proof-of-concept cohort. The majority (*n* = 10, 62.5%) were phase II trials. The mean follow-up period for these trials was 32 (SD 15.04) weeks.

#### 3.1.3. Biologic Drugs

The CTs reported the use of 12 different biological drugs, each with distinct mechanisms of action. The most frequently evaluated biologics in these trials were rituximab (*n* = 4, 25%), with one trial using it in combination with belimumab, which targets the B-cell activating factor (BAFF) while other trials bind this target alone or its receptor (*n* = 4, 25%). Details regarding targeted molecules are provided in Table 1.

### 3.2. Inclusion Criteria and Outcome Measures

With respect to inclusion criteria, a large number of trials focused on patients with high disease activity, as measured by the ESSDAI. Specifically, ten trials required a minimum ESSDAI: nine CTs set the threshold at ESSDAI ≥ 5 and one required an ESSDAI ≥ 6. Dryness was also a common inclusion criterion, using objective and subjective measures. Both SSF and USSF were considered inclusion criteria in seven trials (43.8%), with 42.9% requiring a minimum USSF greater than zero, while the remaining trials utilized stimulated salivary flow with varying thresholds (≥0.1 mL/min in three CTs and ≥0.15 mL/min in one CT). Subjective reports of dryness such as NRS reports or equivalents were considered as inclusion criteria in ten trials (62.5%). An ESSPRI ≥ 5 was required in four trials. All the CTs including the ESSDAI and ESSPRI as part of their eligible criteria were published after 2017. Biomarkers of B-cell activation, such as hypergammaglobulinemia, were included as inclusion criteria in four trials. Seropositivity with a positive anti-Ro/SSA was specified as an admission criterion in five trials.

The primary outcomes in the trials were diverse. Nine CTs (56.3%) focused on the ESSDAI as their primary outcome, with one trial incorporating a composite measure of the ESSDAI and Physician Global Assessment (PhGA) [17], and they were all published after 2017. Improvements in subjective symptoms such as fatigue, dryness, pain, and overall disease impact were the main outcomes in three trials. A reduction in the ESSPRI was the primary outcome in two CTs, both of which were published from 2017 onwards. Changes in SSF were required in two trials. Overall, 50% of the CTs achieved their primary outcomes. Among the nine trials using an ESSDAI score reduction as their primary outcome, four demonstrated a significant improvement, while five failed to meet their primary endpoint. A summary of the inclusion criteria and primary outcome measures is provided in Supplementary Table S1.

### 3.3. Population Characteristics

#### 3.3.1. Demographics

The trials that were reviewed involved a total of 1607 participants, and 89.4% of them completed the studies. Most of the patients (94%) were female, and the mean age was 51.0 (SD 4.04) years. The time since diagnosis was noted in the majority of the CTs (*n* = 14, 87.5%). In 10 trials this was reported as a mean, and among these populations the mean time was 6.47 (SD 2.48) years. Only three CTs recorded the time since symptoms started, with a median of 7.55 (range 6.9, 11.0) years in two of them, and a mean of 7.9 (SD 6.75) in the other one [21].

Race was reported in six (37.5%) trials, and they were all published from 2017 onwards. Four CTs differentiated between White, Asian, and Black patients. One trial only distinguished between White, Black, and “others” [9]. Only one trial collected the data of American Indian and multiracial patients included in the CT (who accounted for two participants in the whole trial) [10]. Among the data available, 77.8% of the participants were White, 11.9% were Asian, and 5.2% were Black. Hispanic or Latino ethnicity was noted in only two (12.5%) trials, and they represented 3.4% of the participants. Other ethnic groups were not considered individually or were collectively categorized as non-Hispanic or non-Latinos.

#### 3.3.2. Immunological and Serological Profiles

Serological profiles of the participants revealed high positivity rates for common markers associated with pSS. Most of the patients (95.6%) were positive for anti-Ro/SSA antibodies. This rate was 92.9% when excluding trials that required anti-Ro/SSA positivity for participation. A considerable proportion of the population (83.1%) tested positive for ANA. Anti-La/SSB and RF were found in 61.2% and 58.7% of patients, respectively. When reported, the mean levels of C3 and C4 were 113.7 (SD 20.9) mg/dL and 21.6 (SD 4.4) mg/dL, while the mean IgG level was 18.4 (SD 4.4) g/L. Reporting of serological markers is summarized in Table 2.

#### 3.3.3. Disease Activity and Manifestations

The ESSDAI was reported in 93.8% of the trials, with a mean value of 9.6 (SD 2.8) and a median value of 10 (range 2.0, 14.0). In terms of objective measurements of glandular impairment, USSF was the most commonly recorded variable (*n* = 14, 87.5%) with a mean rate of 0.104 (SD 0.041) mL/min. Only three trials conducted a salivary gland histological analysis, with 86.1% of the population with a SGFS >1.

Patient-reported outcomes emphasize the symptom burden but were reported inconsistently. The ESSPRI score was the most commonly used (*n* = 15, 93.8%) tool, and the mean value was 6.3 (SD 0.88). The NRS for dryness, fatigue, and pain showed mean values of 7.2/10, 6.7/10, and 5.7/10, respectively. Assessment of the quality of life, physical and mental function, and fatigue with SF-36 and MFI-20 showed mean values of 52.4 (SD 11.5) and 56.4 (SD 4.9), respectively. Complementary information about glandular function, symptom burden, and quality of life scores are shown in Table 3.

Only two trials collected data on the prevalence of lymphoma in the recruited patients, representing 6.7% and 1.8% of the enrolled participants [17,23].

#### 3.3.4. Concomitant Treatment Patterns and Follow-Up

The majority of CTs (*n* = 14, 87.5%) permitted simultaneous treatments. Conventional immunosuppressants (e.g., methotrexate, leflunomide, azathioprine, sulfasalazine, cyclosporin A, and tacrolimus) were allowed in nine (56.3%) CTs, and stable pre-trial dosing was required. Antimalarials (e.g., hydroxychloroquine, chloroquine, and quinacrine) were restricted in four (25%) CTs. Corticosteroids were utilized in 75% of the trials. However, dosing was inconsistently reported, with only three trials specifying the dose of prednisone equivalent, these being 6.02 (SD 3.23) mg/day and 5.6 (SD 2.5) mg/day in the CTs reporting it as a mean, and 6.25 (range 5, 10) mg/day in the CT reporting it as a median. Other trials (37.5%) set a threshold that varied in the range 7.5–15 mg/day. Trial retention rates were robust, with 89.4% of participants completing follow-up periods that averaged 32 weeks.

#### 3.3.5. Geographical Representation

The geographical distribution of the CTs varied. The majority of the studies (*n* = 12, 75%) were multicentric, with an average of 31 (SD 21.9) recruitment centers, while the mean number of countries involved was 7.5 (SD 7.8). Five CTs recruited patients across multiple (≥3) continents, and they were all published after 2017. Half of the trials were conducted entirely in North America or Europe, while 18.8% were exclusively conducted in Asia. None of the CTs included Africa in the recruiting process. The majority of the CTs (*n* = 12, 75%) involved only countries with a very high HDI, whereas two (12.5%) included countries with both high and very high HDIs. Two CTs were conducted separately in regions of China with a high HDI. No recruitment site was located in countries with a medium or low HDI.

## 4. Discussion

The major findings of this systematic review of 16 trials involving biologic treatments of patients with pSS were the limited racial and ethnic diversity of the enrolled participants in the trials that included such a variety of therapeutic targeting, the growing trend towards the utilization of standardized scores (ESSDAI and ESSPRI), and the increasing tendency of conducting multicentric trials with multiple countries and continents involved. However, the latter finding does not always correlate with better racial and ethnic diversity.

**Figure 2:**
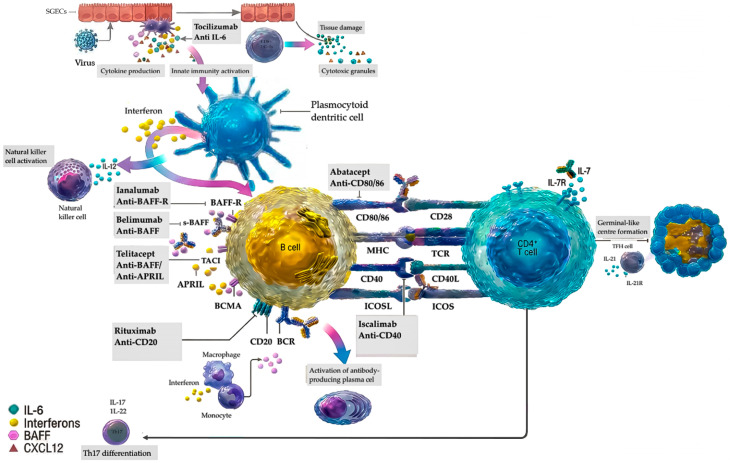
Immune pathways involved in Sjögren syndrome pathophysiology and targets of biologic therapies. In genetically susceptible individuals, viral infection is thought to trigger activation of salivary gland epithelial cells (SGECs), leading to innate immune activation and linking innate immune activation to adaptive immune responses. Activated SGECs can act as antigen-presenting cells and generate a proinflammatory environment characterized by the production of IL-6, type I interferons, CXCL12, B-cell activating factor (BAFF), and a proliferation-inducing ligand (APRIL), thereby promoting immune cell recruitment to the salivary glands and activation of plasmacytoid dendritic cells (pDCs). pDC activation results in increased type I interferon production, further enhancing B-cell activation, mainly through upregulation of BAFF expression. BAFF promotes B-cell survival, maturation, and differentiation predominantly via the BAFF receptor (BAFF-R), and to a lesser extent through TACI and BCMA, while APRIL supports plasma cell survival by acting as a ligand for TACI and BCMA. Activated B cells recognize antigens through the B-cell receptor (BCR) and subsequently activate T cells through a two-step process involving antigen presentation to the T-cell receptor (TCR) as a first signal, followed by a second costimulatory signal mediated by CD80/CD86–CD28, CD40–CD40L, and ICOS–ICOS ligand interactions. CD4^+^ T cells, particularly IL-21-producing T follicular helper cells, play a central role in the formation of germinal center-like structures, where they cooperate with B cells to promote high-affinity autoantibody production. In parallel, epithelial cell damage induced by exogenous triggers and perpetuated by activated CD8^+^ T cells and immune complexes leads to the chronic release of autoantigens, contributing to sustained innate immune activation and disease perpetuation. Additional immune cell populations, including natural killer cells, monocytes and macrophages, innate-like lymphocytes, and T helper 17 cells, further contribute to disease development. Adapted from Baldini et al., Nature Reviews Rheumatology, 2024 [1]. Reprinted with permission.

The biologic agents assessed for pSS target immune pathways relevant to both innate and adaptive immunity. B-cell-directed therapies include depletion of CD20+ cells with rituximab/its biosimilars and inhibition of BAFF-mediated survival signals through agents such as belimumab, ianalumab, and telitacicept. These therapies primarily modulate adaptive immunity by reducing autoreactive B-cell maturation and autoantibody production. Modulation of T-cell activation is another key strategy. Agents such as abatacept and iscalimab disrupt co-stimulatory interactions essential for T–B collaboration, with downstream effects on dendritic-cell and macrophage activation. Additional approaches target cytokine and intracellular signaling pathways. IL-6 blockade with tocilizumab reduces inflammatory signaling, while low-dose interleukin-2 enhances immune regulation. Inhibitors of BTK, remibrutinib, lymphotoxin-β receptor signaling, baminercept, or cathepsin S, RO5459072, are further examples of how biologics influence innate and adaptive compartments by modifying B-cell receptor signaling and antigen processing. These mechanisms highlight the diverse but complementary immune pathways addressed by biologic therapies in pSS, as illustrated in Figure 2, which integrates key immune pathways in Sjögren syndrome pathophysiology with the specific immune targets of biologic therapies [1].

Baseline characteristics among the enrolled patients partially align with established pSS profiles, notably: female predominance (94%), a mean age of 51 years at the time of the study, and high anti-Ro/SSA seropositivity (92.9%) [25,26]. In those trials in which the race of the participants was recorded (37.5%), there was a clear predominance of White patients (77.8%). The Asian and Black populations accounted for 11.9% and 5.2% of the total, respectively, and there was minimal or absent representation of American Indians (<1%) and multiracial (<1%) and Australoid (zero patient reported) populations. Despite known racial and ethnic disparities among pSS patients with respect to prevalence, severity, and treatment response [27,28], 62.5% of trials omitted race and ethnicity data entirely.

Recently the importance of adequate racial and ethnic representation in RCTs has been highlighted because misrepresentation of diverse populations may lead to non-generalizable guidelines. As a result of this growing awareness, several organizations notably the Federal Drug Administration and the United States House of Representatives, have promoted actions targeting this issue [29,30]. It is essential to describe the diversity among patients in CTs thoroughly to assess the efficacy of treatments fully. This is particularly important considering that underrepresented groups often exhibit more severe forms of the diseases, as has been previously documented in other autoimmune rheumatic conditions such as systemic lupus erythematosus (SLE) and systemic sclerosis [31,32].

Geographical biases further exacerbate these issues, as, despite the multicenter design of many of these trials, recruitment was clearly focused on North America and Europe. Recent trials show improved racial reporting and greater diversity in site enrollment, but inclusive recruitment strategies remain essential to ensure that results apply to the broadest possible pSS patient community. It should be noted that no trials included Africa as a recruitment continent or countries with a medium or low HDI. Improving representation from Africa and low-HDI regions will require proactive measures notably funding support for trial infrastructure, the development of international multicenter networks, and the inclusion of diversity requirements in pSS protocol design.

Another noteworthy aspect is the long disease duration among trial participants. The average time since pSS diagnosis exceeded six years. This may explain the high prevalence of irreversible glandular damage or chronic damage (such as gland fibrosis or neuropathy) that may well be linked to an increased burden of subjective symptoms, including pain, fatigue, and brain fog [33]. These persistent and often debilitating symptoms emphasize the critical need for developing effective therapeutic interventions and implementing them earlier in the disease course.

A further striking finding is the substantial heterogeneity in inclusion criteria and primary outcomes. Both showed a dual focus on clinician-assessed and patient-reported measures. The substantial heterogeneity in inclusion criteria and primary outcomes complicates the comparison of the results across studies [34]. Even when using the same parameters, there were variable thresholds (e.g., ESSDAI ≥5 vs. ≥6) and inconsistent cut-offs (e.g., salivary flow 0–0.15 mL/min) that limited cross-trial comparability. Many recent studies include the ESSPRI together with the ESSDAI, recognizing that both objective disease activity and subjective symptom severity are key facets of pSS. The widespread use of the ESSDAI facilitates consistency across studies and reflects its validation as a reliable index of disease activity; the sole exception to its use was a trial that predates the establishment of the ESSDAI. This trend toward standardized activity assessment means that despite the ESSDAI not being the primary endpoint in about half of the trials, it is still collected as a common measure to enhance cross-trial comparisons of disease activity [35]. There is parallel adoption of the ESSPRI for symptom assessment. It was reported in almost 90% of the trials, allowing researchers to more reliably compare the patient-centric outcomes across different trials [36]. However, reliance on subjective endpoints such as ESSPRI and NRS scores for dryness, pain, and fatigue also poses important challenges. These measures are highly susceptible to placebo effects. There is a weak correlation between symptoms and systemic disease activity in pSS. Future trials would benefit from a more standardized use of patient-reported outcomes, including standardized symptom rating scales and agreed thresholds for what constitutes meaningful clinical change, and from systematically combining these measures with objective assessments of glandular function or biomarkers to improve interpretability and cross-trial comparability.

The increasing standardization in the use of the ESSDAI and ESSPRI reflects the maturation of these indices, supported by accumulating validation studies and successive EULAR consensus efforts [35]. This harmonization offers important advantages for future trial design, reducing methodological heterogeneity, facilitating more reliable cross-trial comparisons, and providing a stable foundation for defining consistent response thresholds. This evolution strengthens the methodological infrastructure of pSS trials and supports the establishment of regulatory-grade outcome measures.

This observation aligns with findings from recent reviews of CTs in SS, which have also noted considerable variability in inclusion criteria and outcome measures. However, those reviews included a wider range of interventions such as non-pharmacological and local treatments, and were not specifically focused on systemic pharmacologic therapies, particularly biologic agents [37].

The heterogeneous nature of pSS is insufficiently addressed in current trials. Although the disease can have diverse manifestations and affect multiple organ systems, research persists in employing exclusively broad approaches rather than target therapies to organ-specific manifestations or patient subgroups stratified by biomarker profiles. While a holistic assessment is valuable, this “one-size-fits-all” trial design may overlook improvements that occur in individual domains, as has already been demonstrated in other diseases, such as lupus nephritis and the specific approval of voclosporin [38]. Conducting trials that target a particular organ system could be difficult. However, a stratified trial analysis, where patients from a broadly designed trial are grouped according to the predominant disease domain, could provide insight into whether a drug has a more pronounced effect on one particular manifestation compared to others.

This review benefits from rigorous analysis of contemporary CTs (81.3% published after 2017) and granular demographic and serological data. Nevertheless, this review has some limitations, notably the limited number of trials undertaken and/or published despite 14-year period covered by the analysis. An additional limitation is the inconsistent reporting of baseline serological and demographic variables across trials. These gaps in data availability reduce the comparability of study populations and may obscure meaningful differences between cohorts, potentially contributing to the heterogeneity observed across trials. The publication bias may have also influenced the data available for the review.

## 5. Conclusions

Despite recent improvements in methodological quality, clinical trials of biologic therapies in patients with primary Sjögren’s syndrome continue to exhibit significant underrepresentation of non-White populations. This lack of diversity raises concerns about the generalizability and equity of findings. Additionally, persistent variability in primary endpoints and inconsistent biomarker reporting highlight ongoing challenges in trial comparability and interpretability. Nevertheless, there is growing methodological consensus, particularly in the use of the ESSDAI as a standard tool for assessing systemic disease activity, and increased emphasis on patient-reported outcomes and global trial participation represents encouraging progress. Addressing these disparities remains essential for advancing more inclusive and standardized clinical research in pSS.

## Figures and Tables

**Table 1:** Selected trials for biologics in primary Sjögren’s syndrome.

Authors	Year of Publication	Study Design	Patient Population	Drug Evaluated	Target
Meijer JM et al. [9]	2010	RCT, phase II	30	RTX	CD20
Devauchelle-Pensec V et al. [10]	2014	RCT, phase II/III	120	RTX	CD20
Mariette X et al. [11]	2015	Open-label trial, phase II	30	BEL	BAFF
Bowman SJ et al. [12]	2017	RCT, phase III	133	RTX	CD20
St Clair EW et al. [13]	2018	RCT, phase II	52	Baminercept	LβR
van Nimwegen JF et al. [14]	2020	RCT, phase III	80	Abatacept	CD80/86
Shao Q et al. [15]	2021	RCT, phase II/III	66	Iguratimod	Miscellaneous (TNF-α, IL-1β, IL-6, IL-17, IL-8, IFN-γ)
Baer AN et al. [16]	2021	RCT, phase III	187	Abatacept	CD80/86
Felten R et al. [17]	2021	RCT, phase II/III	110	TCZ	IL-6R
Mariette X et al. [18]	2022	RCT, phase II	86	BEL, RTX	BAFF, CD20
He J et al. [19]	2022	RCT, phase II	60	LD-IL-2	IL-2
Bowman SJ et al. [20]	2022	RCT, phase II	190	Ianalumab	BAFFR
Bentley D et al. [21]	2023	RCT, phase II	75	RO5459072	Cathepsin S
Xu D et al. [22]	2024	RCT, phase II	42	Telitacicept	BAFF, APRIL
Dörner T et al. [23]	2024	RCT, phase II	73	Remibrutinib	BTK
Fisher BA et al. [24]	2024	RCT, phase II	273	Iscalimab	CD-40

RCT = randomized controlled trial; RTX = rituximab; BEL = belimumab; BAFF = B-cell activating factor; LβR = lymphotoxin β receptor; TNF-α = tumor necrosis factor α; IL-1β = interleukin-1β; IL-6 = interleukin-6; IL-17 = interleukin-17; IL-8 = interleukin-8; IFN-γ = interferon-γ; TCZ = tocilizumab; IL-6R = interleukin-6 receptor; LD-IL-2 = low-dose interleukin 2; BAFFR = B-cell activating factor receptor; APRIL = a proliferation-inducing ligand; BTK = Bruton’s tyrosine kinase.

**Table 2:** Serological features of primary Sjögren’s syndrome patients included in the trials.

Serological Features	Statistical Measures	Trials Reporting
ANA positivity (%)	831	4/16
Anti-Ro/SSA positivity (%)	92.9	9/16
Anti-La/SSB positivity (%)	61.2	9/16
RF positivity (%)	58.7	6/16
RF (mean (SD), U/mL)	116.3 (60.1)	3/16
C3 (mean (SD), g/L)	113.7 (20.9)	3/16
C3 (median (range), g/L)	105 (97, 130)	3/16
C4 (mean (SD), g/L)	21.6 (4.4)	6/16
C4 (median (range), g/L)	20 (20, 25)	2/16
IgG (mean (SD), g/L)	18.4 (4.4)	11/16
IgG (median (range), g/L)	20.7 (17.4, 22.9)	2/16
Hypergammaglobulinemia (%)	63.1	3/16
Cryoglobulinemia (%)	10.3	1/16
β2-microglobulin (mean (SD), nmol/L)	245.7 (41.5)	1/16

Antibody positivity, hypergammaglobulinemia, and cryoglobulinemia are reported as percentage of patients expressing the feature among the total patients enrolled in the trials. Trial reporting is expressed as a proportion (*n* of trial reporting the information/total trials). Continuous variables are expressed as mean (SD) or median (min, max). ANA = antinuclear antibodies; anti-SSA/anti-Ro = anti-SSA antibodies against Sjögren’s Syndrome A (anti-SSA) or anti-Ro; anti-SSB/anti-La = antibodies against Sjögren’s Syndrome B (anti-SSB) or anti-La; RF = Rheumatoid Factor; IgG = Immunoglobulin G.

**Table 3:** Sicca symptoms and disease burden measures.

Information Assessed	Statistical Measures	Trials Reporting
Objective measurements of glandular impairment		
SGFS (mean (SD), *n* focus/cm^2^)	2.7 (0.75)	2/16
SGFS > 1 (%)	86.1	2/16
USSF (mean (SD), mL/min)	0.104 (0.041)	11/16
USSF (median (range), mL/min)	0.080 (0.05, 0.10)	2/16
USSF < 0.1 mL/min (%)	69.5	2/16
Subjective measurements and burden of disease		
ESSPRI (mean (SD))	6.3 (0.88)	10/16
ESSPRI (median (range))	6.9 (5.8, 7.3)	3/16
Pain, NRS (mean (SD))	5.7 (0.81)	6/16
Pain, NRS (median (range))	6.5 (4, 7)	3/16
Fatigue, NRS (mean (SD))	6.7 (0.5)	6/16
Fatigue, NRS (median (range))	7 (6, 8)	3/16
Dryness, NRS (mean (SD))	7.2 (0.37)	7/16
Dryness, NRS (median (range))	7 (7, 8)	3/16
SF-36 total (mean (SD))	52.4 (11.5)	2/16
Information assessed		
PCS (mean (SD))	37.8 (1.7)	5/16
PCS (median (range))	50.4 (50.3, 50.4)	1/16
MCS (mean (SD))	40.8 (3.5)	4/16
MCS (median (range))	47 (44, 50.1)	2/16
MFI-20 score (mean (SD))	56.4 (4.949)	1/16
MFI-20 score (median (range))	51.3 (51, 51.5)	1/16

SGFS > 1 and USSF < 0.1 mL/min are reported as the percentage of patients expressing the feature among the total number of patients enrolled in the trials which reported the information. Trial reporting is expressed as a proportion (*n* of trial reporting the information/total trials). Continuous variables are expressed as mean (SD) or median (min, max). SGFS = salivary gland focus score; USSF = unstimulated salivary flow; ESSPRI = EULAR Sjögren’s Syndrome Patient Reported Index; NRS = numerical rating scale; SF-36 = short form 36; MCS = mental component summary; PCS = physical component summary; MFI-20 = multidimensional fatigue inventory 20.

## Data Availability

The data that support the findings of this study are available from the corresponding author upon request.

## Supplementary Materials

The following supporting information can be downloaded at: https://www.mdpi.com/article/10.3390/jcm15030950/s1, Table S1: Summary of inclusion criteria and primary outcomes of the included clinical trials; File S1–S2: PRISMA checklist.
