# Supplementary material for: Comparative Analysis of Clinical Trials of Biologic Drugs for Patients with Primary Sjögren’s Syndrome
Source: J Clin Med. 2026 Jan 24;15(3):950. doi: 10.3390/jcm15030950 (PMC12898365; doi:10.3390/jcm15030950)
Supplement: Supplementary file 1 [file jcm-15-00950-s001.zip › jcm-4015293-supplementary.pdf]

Supplementary Table S1 - Summary of inclusion criteria and primary outcomes of the included clinical trials.

Study	Drug evaluated	Inclusion criteria	Primary outcome variable assessed	Fulfilment of primary outcome
Meijer JM et al., 2010 [9]	Rituximab	Aged ≥18 years old<br>ACR/EULAR SS criteria met<br>Seropositive for RF and anti-Ro/SSA ± anti-La/SSB<br>SSF ≥0.15 mL/min	SSF (mL/min)	Yes
Devauchelle-Pensec V et al., 2014 [10]	Rituximab	Aged 18–80 years old<br>ACR/EULAR 2002 SS criteria met<br>VAS ≥50 mm in ≥2/4 domains (global disease, pain, fatigue, dryness)<br>≤10 years from symptom onset<br>Anti-Ro/SSA or RF positive, B-cell activation marker, extraglandular manifestation, or parotid enlargement<br>Stable steroids dose	VAS for global disease, pain, fatigue, and dryness	No
Mariette X et al., 2015 [11]	Belimumab	ACR/EULAR SS criteria met<br>Anti-Ro/SSA or anti-La/SSB positive<br>≥1 of: B-cell activation biomarker / ≤5 years from symptom onset / systemic complications or persistent salivary gland enlargement	VAS for dryness, fatigue, musculoskeletal pain and systemic activity assessed by the physician.<br>Variation in biomarkers of B cell activation.	Yes
Bowman SJ et al., 2017 [12]	Rituximab	Aged 18–80 years old<br>ACR/EULAR SS criteria met<br>Anti-Ro/SSA positive<br>USSF > 0.0 mL/min<br>Fatigue and oral dryness >5/10 (patient-reported Likert scale)<br>Stable background treatment, including steroids dose	VAS for fatigue and oral dryness.	No
St Clair EW et al., 2018 [13]	Baminercept	Aged 18–75 years old<br>ACR/EULAR 2016 SS criteria met<br>SSF ≥ 0.1 ml/min<br>Severe parotid gland swelling or some systemic disease manifestations<br>Steroids dose ≤ 10 mg/d prednisone or equivalent.	SSF (mL/min)	No
van Nimwegen JF et al., 2020 [14]	Abatacept	Aged ≥18 years old<br>ACR/EULAR 2016 SS criteria met<br>ESSDAI score ≥ 5<br>Positive gland biopsy<br>Time since diagnosis ≤ 7 years	ESSDAI score	No
Shao Q et al., 2021 [15]	Iguratimod	Aged 18–70 years old<br>ACR/EULAR 2002 SS criteria met<br>Symptomatic dry eyes and dry mouth<br>≥1 of: hypergammaglobulinemia; ESR >25 mm/h; ANA/SSA/SSB/RF+	ESSPRI score	Yes
Baer AN et al., 2021 [16]	Abatacept	Aged ≥18 years old<br>ACR/EULAR 2016 SS criteria met<br>ESSDAI score ≥ 5<br>ESSPRI score ≥ 5<br>Anti-Ro/SSA positive<br>Refractory to symptomatic or local therapy	ESSDAI score	No
Felten R et al., 2021 [17]	Tocilizumab	Aged ≥18 years old<br>ACR/EULAR SS criteria met<br>Anti-Ro/SSA positive<br>ESSDAI score ≥ 5	Composite outcome: ESSDAI score, no new moderate/severe ESSDAI	No

		Stable background treatment, steroids dose ≤ 15 mg/d prednisone or equivalent	domain, no PhGA worsening (≥1/10).	
Mariette X et al., 2022 [18]	Belimumab, Rituximab	ACR/EULAR SS criteria met<br>ESSDAI score ≥ 5<br>NRS dryness ≥ 5/10<br>USSF > 0.0 mL/min or SSF > 0.05 mL/min.	Safety	Yes
He J et al., 2022 [19]	Low-dose interleukin 2	Aged 18–70 years old<br>ACR/EULAR 2002 SS criteria met<br>ESSDAI score ≥ 5<br>VAS ≥50 mm in ≥2/3 domains (dryness, pain, fatigue)<br>≥1 of: hypergammaglobulinemia; parotid enlargement; systemic involvement<br>Stable background treatment, steroids dose ≤ 7.5 mg/d prednisone or equivalent	ESSDAI score	Yes
Bowman SJ et al., 2022 [20]	Ianalumab	Aged 18–75 years old<br>ACR/EULAR 2016 SS criteria met<br>ESSDAI score ≥ 6<br>ESSPRI score ≥ 5<br>Anti-Ro/SSA positive<br>SSF ≥ 0.1 mL/min	ESSDAI score	Yes
Bentley D et al., 2023 [21]	RO5459072	Aged 18–75 years old<br>ACR/EULAR 2016 SS criteria met<br>ESSDAI score ≥ 5<br>ESSPRI score ≥ 5<br>Anti-Ro/SSA and or Anti-La/SSB positive	ESSDAI score	No
Xu D et al., 2024 [22]	Telitacicept	Aged 18–65 years old<br>ACR/EULAR 2016 SS criteria met<br>Anti-Ro/SSA positive<br>ESSDAI score ≥ 5	ESSDAI score	No
Dörner T et al., 2024 [23]	Remibrutinib	ACR/EULAR 2016 SS criteria met<br>ESSDAI score ≥ 5<br>ESSPRI score ≥ 5<br>Anti-Ro/SSA positive<br>USSF >0 mL/min	ESSDAI score	Yes
Fisher BA et al., 2024 [24]	Iscalimab	Common criteria:<br>Aged ≥18 years old<br>ACR/EULAR 2016 SS criteria met<br>Anti-Ro/SSA positive<br>SSF ≥ 0.1 mL/min.<br><u>Cohort 1:</u><br>ESSDAI score ≥ 5<br>ESSPRI score ≥ 5<br><u>Cohort 2:</u><br>ESSDAI score <5<br>ESSPRI fatigue or dryness subscale scores of ≥5<br>Impact of Dry Eye on Everyday Life score ≥ 30	Cohort 1: ESSDAI score<br><br>Cohort 2: ESSPRI score	Cohort 1: Yes<br><br>Cohort 2: No

Abbreviations: ACR/EULAR, American College of Rheumatology / European Alliance of Associations for Rheumatology; SS, Sjögren's syndrome; RF, rheumatoid factor; anti-Ro/SSA, anti-Sjögren's-syndrome–related antigen A antibody; anti-La/SSB, anti-Sjögren's-syndrome–related antigen B antibody; SSF, stimulated salivary flow; USSF, unstimulated salivary flow; VAS, visual analogue scale; NRS, numeric rating scale; ESR, erythrocyte sedimentation rate; ANA, antinuclear antibodies; ESSDAI, European Sjögren's Syndrome Disease Activity Index; ESSPRI, European Sjögren's Syndrome Patient-Reported Index.
